# Functional Diversity and Community Composition of Soil Fungi Associated With Canopy Dieback in *Araucaria araucana* Forests of Contrasting Edaphic Conditions

**DOI:** 10.1111/1758-2229.70361

**Published:** 2026-06-23

**Authors:** Valeria Arriagada, Adrian Garrido, Francisca Madariaga, Rodrigo Hasbun, Eugenio Sanfuentes

**Affiliations:** ^1^ Laboratorio de Patología Forestal, Facultad de Ciencias Forestales y Centro de Biotecnología Universidad de Concepción Concepción Chile; ^2^ Laboratorio de Epigenética Vegetal, Departamento de Silvicultura, Facultad de Ciencias Forestales Universidad de Concepción Concepción Chile

**Keywords:** forest conservation, forest decline, fungal microbiome, metabarcoding

## Abstract

**Background:** Soil fungal communities play vital roles in forest ecosystem functioning, yet their relationship with tree health remains insufficiently characterized in many endangered species. **Aims:** This study, investigated the composition and functional structure of soil fungi associated with symptomatic and asymptomatic individuals of *Araucaria araucana* in the Nahuelbuta Coastal Range, Chile. **Materials and Methods:** Using high‐throughput ITS1 sequencing and trait‐based annotation, we compared fungal assemblages across two forest sectors with contrasting edaphic conditions. **Results:** Marked differences in taxonomic and functional composition were observed between sites, with more even and functionally diverse communities in less restrictive soils. Within this site, where both tree health conditions co‐occur, no significant differences in alpha or beta diversity were detected; nevertheless, species‐level and functional guild analyses revealed shifts in composition. A shared core microbiome included taxa putatively identified as endophytes, including *Cladophialophora minutissima*, *Fraxinicola europaea*, *Linnemannia hyalina*, saprotrophs (*Solicoccozyma terricola*, *Helicodendron conglomeratum*, *Pseudogymnoascus roseus*) and a plant pathogen (*Penicillium excelsum*). Symptomatic trees harboured unique stress‐tolerant taxa, including cold‐adapted saprotrophs and ericoid mycorrhizal fungi, while asymptomatic trees supported lignocellulose decomposers, mutualists and early‐successional symbionts. **Discussion:** The observed fungal shifts suggest that canopy dieback is associated with a functional reorganization of the rhizosphere microbiome rather than a simple loss of diversity. **Conclusion:** These findings provide new insights into the fungal ecology of *A. araucana* forest and contribute to understanding how soil microbiomes respond to forest decline under contrasting edaphic conditions.

## Graphical abstract

Soil fungal communities associated with *Araucaria araucana* differ primarily according to site edaphic conditions rather than tree health status. Restrictive, nutrient‐poor soils are linked to higher canopy dieback and distinct functional community shifts, with symptomatic trees harbouring stress‐tolerant taxa and asymptomatic trees supporting decomposers and mutualists.

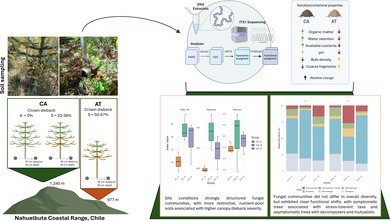

## 1 Introduction

*Araucaria araucana* (Mol.) K. Koch, commonly known as the monkey puzzle tree, is a coniferous species endemic to the temperate forests of Chile and Argentina. Recognized for its remarkable height and longevity, this evergreen tree is among the longest‐lived species globally, with individuals exceeding 1000 years of age (Herrmann 2006; Montaldo Bustos 1974). Its geographic distribution is primarily concentrated in the Andes Mountains, with additional ecologically significant populations found in the Coastal Range (Donoso Zegers and Claudio 1982). As a keystone species, *A. araucana* is vital to in maintaining ecosystem balance, supporting biodiversity, and providing essential habitat and resources for a wide range of flora and fauna. Beyond its ecological contributions, the species holds cultural and economic significance, further underscoring the importance of its conservation. However, due to ongoing habitat degradation, climate change and anthropogenic pressures, *A. araucana* has been classified as Endangered (EN B2ab ii, iii, v) by the International Union for Conservation of Nature (IUCN), indicating significant declines in habitat quality, population size and overall distribution.

The health and vitality of forest trees are intricately linked to their interactions with the soil microbiome, particularly fungal communities within the rhizosphere. A diverse and functionally stable microbiome enhances the resilience of ecological processes to stressors, ensuring the long‐term health of forest ecosystems (Mendes et al. 2015; Tardy et al. 2014). Fungal communities play essential roles in nutrient cycling, organic matter decomposition and plant health. Furthermore, their composition is driven by factors such as soil pH, organic matter content and nutrient availability (Compant et al. 2019). Depending on the specific plant tissue or environment, these communities may include beneficial, neutral or pathogenic microorganisms, which interact in complex ways with their host plants (Hacquard et al. 2017; Trivedi et al. 2020).

In recent years, *A. araucana* populations in the Nahuelbuta Coastal Range have exhibited increasing evidence of forest decline, characterized by crown deterioration (increasing defoliation or loss of crown sectors) and, in severe cases, tree mortality. Symptoms typically begin with chlorosis and yellowing of leaves on the lower branches, progressing upward and outward, ultimately leading to necrosis and branch death. In the Trongol Alto area, for instance, approximately 70% of the forest shows tree mortality, and in some sites, up to 100% of juvenile trees are affected (Sanfuentes et al. 2022). These observations have raised concerns about the factors contributing to the decline, with growing attention directed toward the soil microbiome as a potential driver.

Shifts in soil fungal microbiomes are closely associated with plant health and resilience, as these communities mediate critical processes like nutrient cycling, organic matter decomposition, and stress mitigation (Molina et al. 2020; Morales‐Rodríguez et al. 2024). While some pathogens, such as *Pewenomyces kutranfy* (Balocchi et al. 2021) and *Phytophthora cinnamomi* (Sanfuentes et al. 2022), have been linked to observed symptoms, the complexity of microbial interactions in the soil remains insufficiently elucidated. Furthermore, limited research has been conducted on the soil microbiome of *A. araucana* . To our knowledge, only one published study has examined the endophytic microbiome of *A. araucana* in the context of canopy dieback, yet it lacked differentiation between health conditions or related microbial composition to soil characteristics or tree vitality (Alarcón et al. 2020). This underscores the need for more comprehensive investigations to elucidate the interactions between microbial communities, edaphic factors and host health status.

Recent advances in high‐throughput sequencing (HTS) and the development of functional databases such as FUNGuild and FungalTraits have now enabled insights into the taxonomic identification of soil fungi but also insights into their ecological roles. In forest ecosystems, fungal communities encompass a wide range of functional groups—including pathogens, mutualists and decomposers—that contribute to plant health and ecosystem functioning. Considering the canopy dieback observed in *A. araucana* forests, understanding the composition and relative contribution of these ecological guilds may provide critical insights into potential disease‐promoting or disease‐buffering interactions in the rhizosphere. In this context, profiling the functional structure of soil fungal communities can help contextualize the presence of *P. cinnamomi* within a broader ecological framework.

In this study, we examine the composition and functional roles of fungal communities in soils associated with symptomatic and asymptomatic *A. araucana* trees in the Nahuelbuta Coastal Range. Based on previously documented edaphic contrasts between two representative forest sectors—Antena (severely affected) and Carampangue (moderately affected) (Arriagada, in prep.)—we investigate whether these environmental differences, which have been associated with increased canopy dieback, also structure the soil fungal microbiome. Specifically, we aim to (i) characterize fungal diversity across tree health categories, (ii) identify fungal taxa potentially linked to canopy dieback or resilience and (iii) evaluate how fungal functional guilds respond to edaphic conditions previously shown to differ significantly between sites. Understanding these patterns will contribute to a more comprehensive view of soil ecosystem processes in *A. araucana* forests under decline.

## 2 Methods

### 2.1 Study Area

This study was conducted in the Nahuelbuta Coastal Range, south‐central Chile, at two distinct forest sectors: Antena (AT) and Carampangue (CA), located at coordinates 37°35′ S, 73°10′ W (AT) and 37°42′ S, 73°10′ W (CA), respectively. These sites were selected based on prior evidence of contrasting levels of canopy dieback in *Araucaria araucana* forests, with AT showing more severe symptoms compared to CA (Sanfuentes et al. 2022). The surrounding forest is part of the temperate resinous forest zone, where *A. araucana coexists with native species* such as *Nothofagus dombeyi* , *Gevuina avellana*, *Lomatia hirsuta* and *Escallonia rubra* .

### 2.2 Soil Sampling and Experimental Design

Fungal community analyses were based on soil samples collected beneath the crowns of *A. araucana* trees. In AT, samples were collected from three symptomatic trees. In CA, three symptomatic and three asymptomatic trees were sampled. For each tree, two soil cores were collected at a depth of 50 cm and at 30 cm distance from the trunk (north and south orientations) and subsequently pooled into a single composite sample. All samples were transported to the laboratory and stored at −20°C until processing. Three independent soil samples were collected per condition to ensure biological replication while maintaining comparable sampling depth across sites. This replication level is consistent with standard practices in environmental microbiome studies, providing sufficient statistical power to capture dominant community trends and assess within‐group variability.

Soil physicochemical properties from the same sampling sites were characterized in a previous study (Arriagada, in prep.) and revealed marked edaphic contrasts between sectors. Soils in AT, where canopy dieback symptoms were more severe, exhibited higher bulk density (surface: 0.80 g cm^−3^; 30 cm: 1.08 g cm^−3^), greater coarse fragment content (29%), lower organic matter content across depths (surface: 12.4%; 30 cm: 7.5%; weathered organic material: 3.7%), lower available water‐holding capacity (38.1 × 10^6^) and more acidic conditions (SpH = 4.8; WpH = 5.2), together with reduced nutrient availability, particularly phosphorus (Olsen *p* = 5.91 mg kg^−1^) and boron (0.96 mg kg^−1^). In contrast, CA soils showed lower bulk density (surface: 0.76 g cm^−3^; 30 cm: 0.90 g cm^−3^), lower coarse fragment content (17.6%), higher organic matter content (surface: 18.5%; 30 cm: 14.2%; weathered organic material: 9.0%), higher water‐holding capacity (79.4 × 10^6^), slightly higher pH (SpH = 5.11) and greater phosphorus availability (Olsen *p* = 7.04 mg kg^−1^). These data were not reanalyzed here but were used to contextualize patterns in fungal community composition.

### 2.3 DNA Extraction, PCR Amplification and Sequencing

DNA was extracted from the fine soil fraction using the DNeasy PowerSoil Kit (Qiagen) at the Laboratorio de Patología Forestal, Facultad de Ciencias Forestales, Universidad de Concepción. Amplicon libraries were generated following the Illumina 16S Metagenomic Sequencing Library Preparation protocol. The fungal ITS region was amplified using the ITS1 (5′‐CTTGGTCATTTAGAGGAAGTAA‐3′) and ITS2 (5′‐GCTGCGTTCTTCATCGATGC‐3′) primers, each tagged with unique barcodes. PCR was performed using the Platinum SuperFi II Master Mix (Thermo Fisher Scientific) with the following cycling conditions: 98°C for 30 s; 35 cycles of 98°C for 10 s, 60°C for 10 s and 72°C for 30 s; followed by a final extension at 72°C for 5 min. Negative controls showed no detectable amplification. Sequencing was carried out at Austral‐Omics (Valdivia, Chile) on an Illumina NextSeq platform (2 × 300 bp). Raw data are available under BioProject accession number PRJNA1242245.

### 2.4 Sequence Processing and Taxonomic Assignment

Sequence data were processed using QIIME 2 (Ruiz Gómez et al. 2019). Quality filtering, denoising, chimera removal and ASV inference were performed using the DADA2 plugin. Rarefaction was applied to standardize sequencing depth across samples, setting a threshold of 75,000 reads per sample. Samples below this threshold were excluded from diversity analyses (Table S1).

Taxonomic assignment was performed using the UNITE dynamic database (version 10, QIIME release February 2025). Taxonomic identities were assigned based on sequence similarity and classifier confidence scores, and only assignments with a consensus value of 1.0 were retained to ensure high‐confidence annotation. These assignments generally corresponded to high sequence similarity (typically ≥ 97%), although species‐level identifications should be interpreted as putative due to the limited resolution of ITS1 metabarcoding.

To identify core and unique fungal species across groups, amplicon sequence variant (ASV) data were transformed into presence/absence matrices at the species level. A species was defined as core within a given group when it was detected in all samples of that group. Species were classified as general core taxa if they were present in all samples across all groups. Conversely, unique taxa were those detected exclusively in one group and completely absent in all others. These criteria were implemented using custom R scripts based on group‐wise aggregation and comparative filtering of presence/absence patterns.

### 2.5 Functional Guild Classification

Functional assignment of fungal ASVs was performed using the FUNGuild database (Nguyen et al. 2016), which assigns taxa to ecological guilds based on curated keyword matches. To enhance ecological interpretation and align the analysis with the study objectives, we applied a custom categorization scheme based on six functionally and ecologically relevant groups. Each ASV was assigned to one of these groups according to detected keywords in the ‘Guild’ field and based on a hierarchy of ecological relevance to *A. araucana* canopy dieback.

The first priority category was Plant Pathogen, which included ASVs annotated as ‘plant pathogen’, ‘root pathogen’ or generically as ‘pathogen’ (excluding animal‐related terms). These taxa were considered the most ecologically significant, as they represent potential biotic stressors directly linked to the observed decline symptoms. The second category, Ectomycorrhizal fungi, included terms such as ‘ectomycorrhizal’, ‘ericoid mycorrhizal’ or ‘orchid mycorrhizal’, reflecting their role as dominant mutualists in temperate forests. The third group, Endophytes, encompassed ASVs labelled as ‘endophyte’, ‘foliar endophyte’ or ‘root endophyte’, which may act as mutualists or latent pathogens and are increasingly recognized as transitional players in the plant–microbe continuum.

The fourth category, Plant Saprotrophs, grouped taxa involved in organic matter decomposition and nutrient cycling. It included keywords such as ‘plant saprotroph’, ‘wood saprotroph’, ‘litter saprotroph’, ‘soil saprotroph’ and ‘undefined saprotroph’. Fifth, we retained a category labelled Other Symbionts/Parasites, comprising ASVs identified as ‘fungal parasite’, ‘lichenized’, ‘bryophyte parasite’, ‘epiphyte’ or ‘animal pathogen’. Although less directly relevant to our focal system, these groups were retained to preserve ecological coherence and dataset completeness. Lastly, ASVs with ambiguous annotations or without clear ecological classification were placed in the Unassigned category, to ensure traceability and support further exploratory analyses.

## 3 Results

### 3.1 Taxonomy and Community Composition

The bioinformatic analysis with QIIME 2 yielded a total of 2523 amplicon sequence variants (ASVs). Of these, 147 were assigned at the species level and 640 at the genus level, providing ecologically relevant insights into fungal taxonomic identity despite the limited resolution typically associated with environmental ITS1 datasets.

**FIGURE 1:**
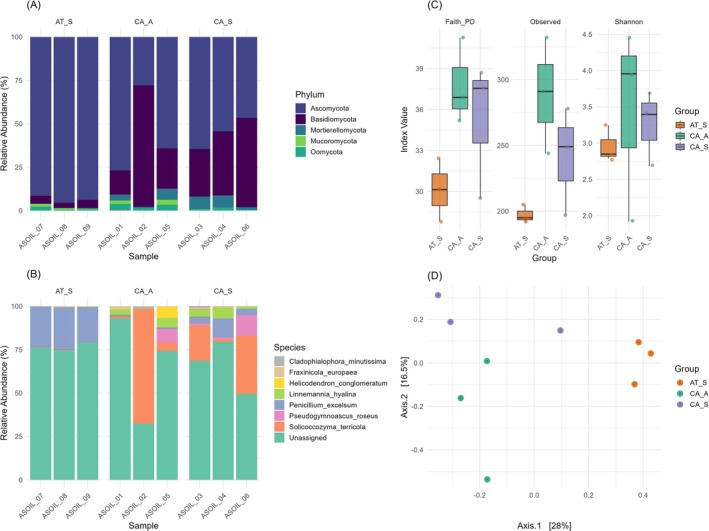
Fungal community composition and diversity across *A. araucana* forest sectors with contrasting edaphic conditions. (A) Relative abundance of dominant fungal phyla, with the top five phyla ranked by overall relative abundance across samples (remaining taxa grouped as ‘others’) in individual soil samples from the restrictive (AT) and less restrictive (CA) sites, categorized by tree health status (symptomatic (S), asymptomatic (A)); (B) relative abundance of the most frequent species, with the top eight species ranked by overall relative abundance across all samples (remaining taxa grouped as ‘others’) in the same samples; (C) Alpha diversity indices: Faith's Phylogenetic Diversity (PD), Observed ASVs and Shannon index across sample groups; (D) Principal Coordinates Analysis (PCoA) based on Bray–Curtis dissimilarity showing clustering by site and tree health category.

At the phylum level, fungal community composition diverged markedly between sites with contrasting edaphic conditions. In the restrictive site, previously characterized by lower nutrient availability, higher soil compaction and reduced water‐holding capacity (Arriagada, in prep.), the community was overwhelmingly dominated by Ascomycota, which represented 93.6% of the total relative abundance. In contrast, the less restrictive site exhibited a more equitable phylum distribution. The stacked bar plot (Figure 1A) illustrates shifts in the relative abundance of dominant fungal phyla across soil samples, with some showing persistent Ascomycota dominance, while others display a greater contribution from Basidiomycota and Mortierellomycota. This within‐group variability suggests that, beyond site‐level edaphic contrasts, microenvironmental heterogeneity and stochastic processes may influence fungal community composition, even under comparable environmental and physiological conditions.

At the species level, community composition also varied notably among groups (Figure 1B). Samples from the restrictive site (AT) were dominated by a small number of ASVs, particularly from the genus Cladophialophora, whereas samples from the less restrictive site (CA) exhibited higher species diversity. In these samples, taxa putatively assigned to *Solicoccozyma terricola*, *Linnemannia hyalina*, *Pseudogymnoascus roseus*, *Penicillium excelsum* and *Fraxinicola europaea* were relatively abundant, especially in asymptomatic individuals.

Alpha diversity patterns, evaluated using Observed ASVs, Shannon index, and Faith's Phylogenetic Diversity (PD), showed differences in diversity across conditions (Figure 1C). While Shannon and Faith's PD indices did not differ significantly among groups (Kruskal–Wallis, *p* = 0.670 and *p* = 0.177, respectively), Observed richness was higher in the less restrictive site (CA), particularly among asymptomatic trees, with marginal significance (Kruskal–Wallis χ^2^ = 5.07, df = 2, *p* = 0.079). These patterns indicate a trend toward greater taxonomic richness in less stressful soil environments, although variability within groups may obscure statistical significance.

Beta diversity analysis using Bray–Curtis dissimilarity (Figure 1D) confirmed a clear separation between fungal communities associated with the restrictive (AT) and less restrictive sites (CA). Samples clustered by site and, to a lesser extent, by tree health status. This pattern was confirmed by PERMANOVA, which showed that grouping (restrictive vs. less restrictive site and tree health condition) explained a significant proportion of the variation in fungal community composition (R^2^ = 0.408, *p* = 0.006).

Given the strong differences observed between sites and the previously established influence of edaphic conditions on fungal community composition (Arriagada, in prep.), subsequent analyses focused exclusively on the less restrictive site (CA), where both symptomatic and asymptomatic trees were sampled. This approach allowed us to better assess whether tree health status was associated with shifts in fungal composition, functional structure or species‐level representation under more ecologically permissive conditions.

### 3.2 Functional Guilds and Species‐Level Patterns in the Less Restrictive Site (CA)

To evaluate whether fungal community composition reflects tree health status under comparable environmental conditions, we analysed fungal assemblages at the less restrictive site (CA), where both symptomatic (S) and asymptomatic (A) *A. araucana* individuals were sampled.

**FIGURE 2:**
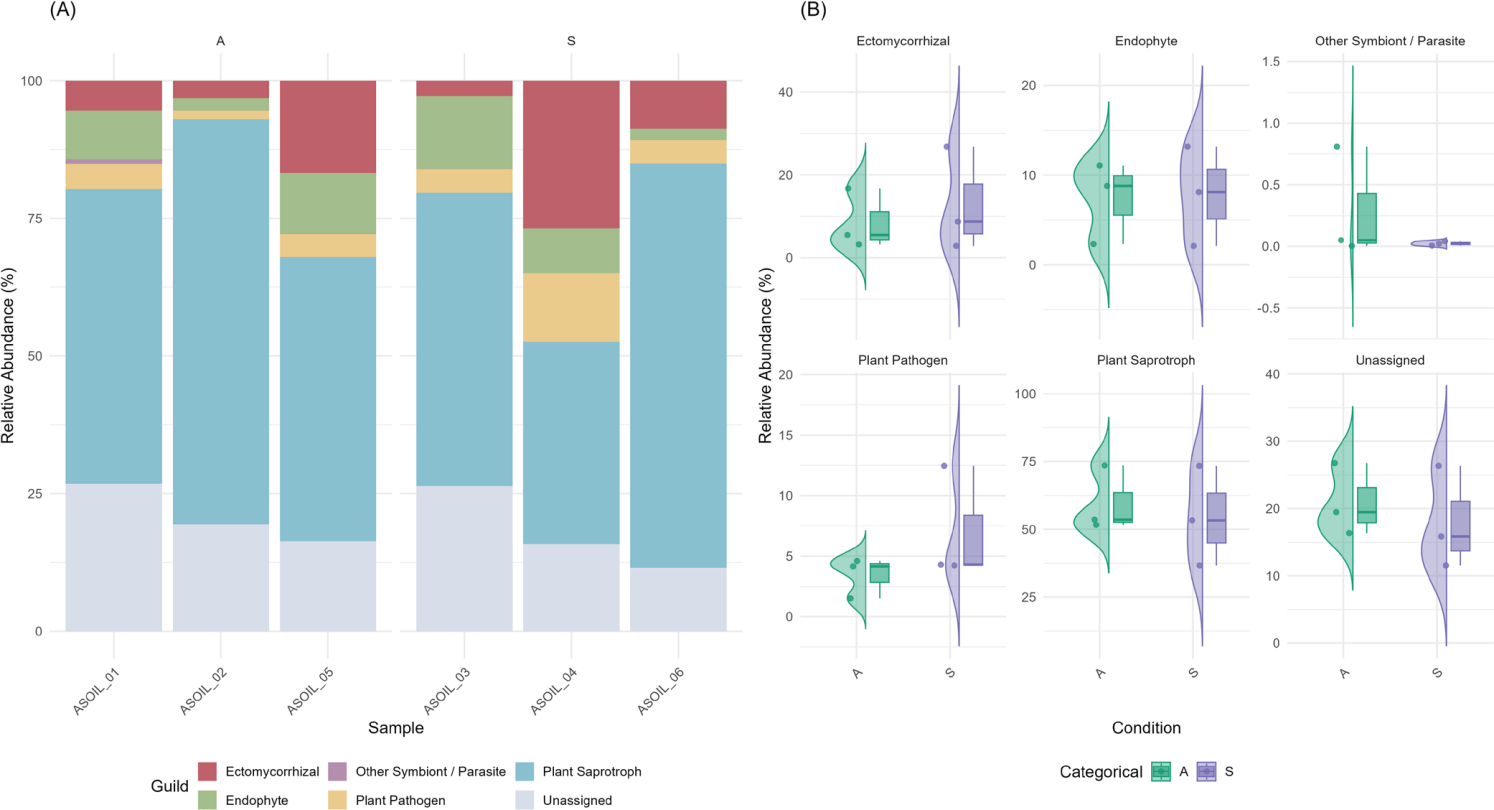
Relative abundance of fungal ecological guilds in rhizospheric soils from asymptomatic (A) and symptomatic (S) *A. araucana* trees at the CA site. (A) Stacked barplots showing the guild composition per sample. (B) Raincloud plots displaying the distribution of guild‐level relative abundances by tree condition. No significant differences were detected between groups (Wilcoxon rank‐sum tests, all p.adj ≥ 0.05).

At this site, fungal communities were dominated by plant saprotrophs, which accounted for the highest mean relative abundance in both asymptomatic (59.6%) and symptomatic trees (54.5%). Other prominent guilds included ectomycorrhizal fungi (8.5% in A vs. 12.7% in S), endophytes (7.4% in A vs. 7.8% in S) and plant pathogens (3.4% in A vs. 7.1% in S). Minor contributions were observed from other symbionts or parasites (< 0.3%) and from unassigned taxa, which represented 20.9% and 17.9% of the total community in A and S trees, respectively (Figure 2A).

Despite these differences in averages, the results showed no significant differences in proportions in the relative abundance of these guilds between tree health categories (Wilcoxon rank‐sum tests, all p.adj = 1.0; Figure 2B). Likewise, species‐level richness and beta diversity did not differ significantly between symptomatic and asymptomatic trees, as confirmed by PERMANOVA (R^2^ = 0.229, *p* = 0.2).

A subset of 20 fungal species was identified as ecologically relevant based on their distribution across tree health categories at the CA site. Seven of these species were consistently detected in both symptomatic and asymptomatic individuals, constituting a conservative core microbiome (Table 1). This group included three endophytes (*Cladophialophora minutissima*, *Fraxinicola europaea*, *Linnemannia hyalina*), three plant saprotrophs (*Solicoccozyma terricola*, *Helicodendron conglomeratum*, *Pseudogymnoascus roseus*) and one plant pathogen (*Penicillium excelsum*).

**TABLE 1:** Core and condition‐specific fungal species detected in rhizospheric soils of *A. araucana* trees at the CA site.

Species	Group	Relative abundance (%)		Functional guild	Reported in literature			Putative ecological role	References
		A	S		Araucaria	Others conifers	Others trees		
Core taxa (shared between A and S)									
*Cladophialophora minutissima*	Core	0.231	0.105	Endophyte		✓		Nutrient mobilization; stress tolerance in conifer soils	Davey and Currah 2007; Hua et al. 2024; Toju and Sato 2018
*Fraxinicola europaea*	Core	0.018	0.013	Endophyte			✓	Common in regenerating forests; may structure microbial communities	Bakys et al. 2022
*Linnemannia hyalina*	Core	0.071	0.093	Endophyte		✓		Soil stabilizer in alpine/subalpine ecosystems	Obase et al. 2023; Telagathoti et al. 2022
*Solicoccozyma terricola*	Core	1.403	1.085	Plant Saprotroph			✓	Dominant yeast in forest soils; sensitive to pH and land use	Klavina et al. 2022; Mašínová et al. 2017
*Helicodendron conglomeratum*	Core	0.718	0.026	Plant saprotroph		✓	✓	Litter decomposer; co‐occurs with ectomycorrhizal fungi	Kwaśna et al. 2021; Tyub et al. 2018; Zhang et al. 2019
*Pseudogymnoascus roseus*	Core	0.544	0.947	Plant saprotroph		✓		Cold‐adapted decomposer in organic‐rich forest soils	Malewski et al. 2023; Naumova et al. 2022
*Penicillium excelsum*	Core	0.268	2.998	Plant pathogen			✓	Nutrient cycler; some strains may promote plant growth	Hou et al. 2024; Klavina et al. 2022; Park et al. 2020; Yang et al. 2017
Unique to asymptomatic trees (A)									
*Byssocorticium atrovirens*	Unique	0.017	—	Ectomycorrhizal		✓		Enhances nutrient uptake and drought tolerance	Karasiński et al. 2009; Shi et al. 2002
*Distoseptispora effusa*	Unique	0.010	—	Plant saprotroph			✓	Lignocellulose decomposer	Karimi et al. 2024; Liao et al. 2025
*Myrmecridium juncicola*	Unique	0.019	—	Plant saprotroph		✓	✓	Wood and litter decomposer in temperate forests	Kwaśna et al. 2016
*Parafusicladium intermedium*	Unique	0.004	—	Plant saprotroph			✓	Involved in organic matter turnover	Shen et al. 2020
*Rhodosporidiobolus colostri*	Unique	0.0005	—	Plant saprotroph		✓		Cold‐tolerant lignin‐degrading yeast	(Margesin et al. 2022; Pan et al. 2024)
*Podospora minicauda*	Unique	0.041	—	Endophyte			✓	Endophytic decomposer contributing to nutrient dynamics	Li et al. 2019; Zhang et al. 2021
*Placynthiella oligotropha*	Unique	0.011	—	Other symbiont/parasite		✓		Forms biological soil crusts in early‐successional forests	Stefańska‐Krzaczek and Szymura, n.d.; Yatsyna 2011
Unique to symptomatic trees (S)									
*Amauroascus albicans*	Unique	—	0.002	Plant saprotroph		✓		Organic matter decomposer; adaptable to diverse habitats	Piontelli et al. 1990; Torres‐Garcia et al. 2023
*Amylocorticiellum molle*	Unique	—	0.006	Plant saprotroph		✓		Brown‐rot fungus on conifer wood	Gates et al. 2021; Gorjón et al. 2011
*Cuphophyllus adonis*	Unique	—	0.005	Plant saprotroph		✓		Found in acidic soils; potential ecosystem indicator	Halbwachs et al. 2018; Voitk et al. 2020; Zhai et al. 2023
*Hyaloscypha variabilis*	Unique	—	0.059	Ectomycorrhizal		✓		Forms ericoid mycorrhiza; contributes to organic matter breakdown	Vohník et al. 2022
*Naganishia diffluens*	Unique	—	0.030	Plant saprotroph			✓	Psychrotolerant yeast interacting with mycorrhizal fungi	Mestre and Fontenla, n.d.; Nilsen et al. 2020; Sannino et al. 2021
*Piskurozyma capsuligena*	Unique	—	0.020	Plant saprotroph		✓		Root‐associated saprotroph; possibly linked to mycorrhizal networks	Mašínová et al. 2017; Mestre and Fontenla, n.d.

*Note:* Relative abundance (%) refers to the mean proportion of each species across asymptomatic (A) and symptomatic (S) individuals. Functional guilds were assigned based on FUNGuild databases. Literature records indicate prior associations with *A. araucana* , other conifers or non‐coniferous tree species. Ecological roles were compiled from published sources and reflect putative functions in forest soils.

Notable differences in relative abundance were observed among core taxa. *H. conglomeratum* was more abundant in asymptomatic trees (0.72%) than in symptomatic ones (0.03%), whereas *P. excelsum* showed the opposite pattern, with higher abundance in symptomatic individuals (2.99%) compared to asymptomatic ones (0.27%). *P. roseus* was also more abundant in symptomatic trees (0.95%) than in asymptomatic ones (0.54%). The remaining core species showed relatively balanced distributions across conditions.

In addition to shared species, six taxa were exclusive to symptomatic individuals: *Amauroascus albicans*, *Amylocorticiellum molle*, *Cuphophyllus adonis*, *Naganishia diffluens*, *Piskurozyma capsuligena* and *Hyaloscypha variabilis*. These were primarily classified as plant saprotrophs or ectomycorrhizal fungi.

Conversely, eight species were exclusive to asymptomatic individuals, including the ectomycorrhizal fungus *Byssocorticium atrovirens*, several plant saprotrophs (*Distoseptispora effusa*, *Myrmecridium juncicola*, *Parafusicladium intermedium*, *Rhodosporidiobolus colostri*), one endophyte (*Podospora minicauda*) and a lichenized symbiont (*Placynthiella oligotropha*).

## 4 Discussion

This study highlights the taxonomic and functional complexity of soil fungal communities associated with *Araucaria araucana* , with a strategic focus on the less restrictive site (CA), where both symptomatic and asymptomatic trees coexist under similar environmental conditions. Through integrating community composition, functional guilds and species‐level resolution, our findings provide early insights into fungal dynamics potentially linked to tree health status.

While alpha and beta diversity metrics did not differ significantly between symptomatic and asymptomatic trees, analyses at the taxonomic and guild level revealed consistent ecological shifts.

Although the sampling design allowed for a controlled comparison across sites and tree conditions, the relatively low number of biological replicates (*n* = 3 per condition) may limit the statistical power to detect subtle differences in alpha and beta diversity. This constraint is particularly relevant in microbial ecology studies, where high within‐group variability is common. Therefore, the absence of significant differences in diversity metrics should be interpreted with caution, as it may reflect limited sensitivity rather than true ecological equivalence.

A core microbiome of seven species, shared across both conditions, included functionally diverse taxa: endophytes (*Cladophialophora minutissima*, *Fraxinicola europaea*, *Linnemannia hyalina*), saprotrophs (*Solicoccozyma terricola*, *Helicodendron conglomeratum*, *Pseudogymnoascus roseus*) and the putative pathogen *Penicillium excelsum*. These taxa have been previously associated with organic matter turnover, nutrient cycling and stress mitigation in forest soils (Davey and Currah 2007; Morales‐Rodríguez et al. 2024; Obase et al. 2023).

While species‐level patterns provide useful ecological insights, these interpretations should be considered in light of the taxonomic resolution limitations inherent to ITS1 metabarcoding.

However, differences in relative abundance within core taxa suggest that similar presence does not imply functional parity. The taxon assigned to *P. excelsum* was approximately ten times more abundant in symptomatic trees (2.99%) compared to asymptomatic ones (0.27%), while *P. roseus*, a cold‐adapted decomposer commonly found in organic‐rich soils (Malewski et al. 2023; Naumova et al. 2022), was also more abundant in symptomatic trees (0.95% vs. 0.54%). By contrast, *H. conglomeratum*, a saprotroph known to co‐occur with ectomycorrhizal fungi in forest soils (Kwaśna et al. 2021; Tyub et al. 2018; Zhang et al. 2019), showed a contrasting pattern, being 27 times more abundant in asymptomatic individuals. These shifts may reflect early changes in rhizosphere function associated with host physiological decline or resource availability related to host decline.

Additionally, exclusive species further distinguished the two tree conditions. Symptomatic individuals hosted stress‐tolerant saprotrophs and yeasts such as *Naganishia diffluens*, *Amauroascus albicans* and *Piskurozyma capsuligena*, all previously reported from cold, organic‐rich or disturbed environments (Mestre and Fontenla, n.d.; Nilsen et al. 2020; Sannino et al. 2021; Torres‐Garcia et al. 2023), as well as the ericoid mycorrhizal *Hyaloscypha variabilis*, typically associated with acidic soils and organic matter breakdown (Vohník et al. 2022). These taxa may be favoured under altered root exudation profiles associated with host physiological decline, signalling a microbial shift toward opportunistic decomposers that exploit weakened hosts.

In contrast, asymptomatic trees supported a broader functional profile, including *Byssocorticium atrovirens*, an ectomycorrhizal fungus known to enhance nutrient uptake and drought resilience in conifers (Karasiński et al. 2009; Shi et al. 2002), and lignocellulose‐degrading saprotrophs such as *Distoseptispora effusa* and *Myrmecridium juncicola*, both associated with wood and litter decomposition in temperate forests (Kwaśna et al. 2016). The presence of *Placynthiella oligotropha*, a lichenized symbiont forming soil crusts in open‐canopy forests (Stefańska‐Krzaczek and Szymura, n.d.; Yatsyna 2011), suggests additional stability and microbial buffering in these rhizospheres.

These compositional differences align with the hypothesis of functional reorganization, where community shifts are driven not merely by species loss but by the replacement of mutualistic and functionally stable taxa with stress‐adapted saprotrophs and opportunistic symbionts. Similar transitions have been documented in temperate forest systems undergoing decline, where reductions in host vitality are accompanied by an increase in decomposer activity and a decline in mycorrhizal or endophytic partners (Azim Nejad et al. 2021; Jankowiak et al. 2024; Schmied et al. 2023; Venice et al. 2021). In these systems, drought and soil degradation act as predisposing stressors, weakening hydraulic performance and nutrient balance, thereby promoting the establishment of fungal taxa capable of exploiting carbon‐limited or decaying tissues. Moreover, as highlighted by García‐García et al. (2023), microsite variability in soil moisture and nutrient availability can modulate such responses at fine spatial scales, suggesting that the patterns observed in *A. araucana* soils reflect broader mechanisms linking edaphic heterogeneity, physiological decline and microbial restructuring in forest dieback contexts.

This functional reorganization is characterized by a shift in ecological strategies and potential metabolic capabilities of the dominant fungal taxa. Fungi exclusive to asymptomatic trees were largely composed of lignocellulose‐degrading saprotrophs (*Distoseptispora effusa*, *Myrmecridium juncicola*, *Rhodosporidiobolus colostri*); (Karimi et al. 2024; Kwaśna et al. 2016; Margesin et al. 2022; Pan et al. 2024) and ectomycorrhizal or endophytic partners such as *Byssocorticium atrovirens* and *Podospora minicauda* (Karasiński et al. 2009; Li et al. 2019; Shi et al. 2002; Zhang et al. 2021), suggesting a rhizosphere structured around complex carbon turnover, nutrient acquisition and mutualistic interactions. In contrast, symptomatic individuals harboured fungal taxa commonly associated with physiological stress or senescent tissues, including psychrotolerant yeasts (*Naganishia diffluens*, *Piskurozyma capsuligena*; Mašínová et al. 2017; Mestre and Fontenla, n.d.; Nilsen et al. 2020; Sannino et al. 2021) and opportunistic decomposers (*Amauroascus albicans*, *Amylocorticiellum molle*; Gates et al. 2021; Gorjón et al. 2011; Torres‐Garcia et al. 2023). These taxa may be favoured under conditions of reduced root activity, altered rhizodeposition and edaphic stress—such as increased compaction and acidity—characteristic of the AT site. The higher abundance of *Penicillium excelsum*—a potential pathogen and nutrient cycler—in symptomatic trees further supports the idea of a rhizosphere increasingly dominated by fungi capable of exploiting weakened hosts (Hou et al. 2024; Klavina et al. 2022; Park et al. 2020; Yang et al. 2017). Together, these patterns indicate a reorganization of ecological functions in the rhizosphere, with likely consequences for carbon use efficiency, nutrient cycling and tree–microbe interactions under decline.

Critically, the fungal shifts observed in symptomatic *A. araucana* individuals may not be purely reactive but rather part of a broader process of physiological predisposition. As proposed by Puchi et al. (2021), drought‐induced mortality in this species may result from a prolonged trajectory of hydraulic failure and growth suppression, exacerbated by the isohydric behaviour of *A. araucana* . This conservative water‐use strategy minimizes water loss but reduces carbon assimilation under drought, increasing vulnerability to carbon starvation and impairing investment in structural defences. (Puchi et al. 2021) reported thinner cell walls in declining trees, suggesting depleted carbon pools and structural weakening.

Such predisposing conditions could open ecological niches for opportunistic or latent fungi. Taxa exclusive to symptomatic individuals—such as *H. variabilis* , *N. diffluens* and *P. capsuligena*—may exploit these weakened tissues or respond to altered rhizodeposition patterns. Their enrichment is likely non‐incidental but rather reflective of microbial opportunism in a host under chronic abiotic stress. These findings support a multifactorial model of decline, where environmental stress interacts with microbial community turnover to reinforce tree deterioration.

Importantly, the clear phylum‐level divergence between sites adds a second dimension of environmental filtering. In the restrictive AT site, fungal communities were overwhelmingly dominated by Ascomycota (~94%), with lower diversity and functional redundancy, likely reflecting edaphic limitations (Arriagada, in prep.). In contrast, the CA site hosted higher diversity and a more balanced phylum representation, offering a more suitable context to disentangle fungal responses to tree health.

While this study is exploratory due to its limited sample size, the consistency of ecological signals across taxonomic, functional and guild dimensions highlights its contribution. Several species reported here—such as *P. excelsum* , *B. atrovirens* and *R. colostri* —have not previously been documented in *A. araucana* forests, underscoring the novelty of the dataset. Moreover, the trait‐based ecological framework adopted here allows for a nuanced understanding of microbial community function beyond mere composition.

This work sets the stage for more targeted studies involving fungal isolation, colonization assays and host physiological monitoring. In an era of increasing environmental pressures and emerging forest decline, advancing our understanding of soil microbial ecology will be essential to protect long‐lived conifers like *A. araucana* , particularly those restricted to fragmented or vulnerable habitats.

## 5 Concluding Remarks and Prospects

This study reveals that *A. araucana* trees growing under comparable edaphic conditions but differing in health status host distinct fungal communities at the species and guild level. Although global diversity metrics did not vary significantly, asymptomatic trees tended to associate with a more functionally diverse set of fungal taxa, including mutualists and decomposers linked to rhizosphere resilience. In contrast, symptomatic individuals were characterized by a higher abundance of stress‐adapted saprotrophs and taxa commonly associated with disturbed or declining environments.

Importantly, these shifts occurred in the absence of major compositional changes at the phylum or guild level, indicating that microbial responses to host decline are better explained by functional reorganization—marked by a shift from mutualistic and lignocellulose‐degrading taxa to opportunistic, stress‐tolerant saprotrophs—than by outright species loss. The identification of core species consistently present across tree conditions also highlights potentially foundational fungi that may sustain soil processes regardless of host health.

While exploratory due to sample size constraints, this work provides novel insights into the fungal ecology of *A. araucana* forests and lays the groundwork for future investigations. Follow‐up studies incorporating temporal sampling, root colonization data and functional assays will be essential to clarify the ecological roles of key fungal taxa. Such knowledge is crucial for informing conservation strategies for this endangered conifer, particularly under ongoing climate and environmental pressures.

Future research should focus on isolating key fungal taxa and conducting controlled colonization assays to determine their functional roles and potential contributions to host health or decline. Integrating experimental approaches with longitudinal field studies will be essential to disentangle causal relationships within the *A. araucana* rhizosphere microbiome.

## Author Contributions

**Valeria Arriagada:** conceptualization, investigation, funding acquisition, writing – original draft, methodology, writing – review and editing, software, formal analysis, project administration, data curation. **Adrian Garrido:** methodology. **Francisca Madariaga:** writing – review and editing. **Rodrigo Hasbun:** conceptualization, methodology, supervision. **Eugenio Sanfuentes:** conceptualization, methodology, supervision.

## Ethics Statement

The authors have nothing to report.

## Consent

The authors have nothing to report.

## Conflicts of Interest

The authors declare no conflicts of interest.

## Supporting information


**Table S1:** Summary of sequencing read processing and quality filtering for all samples.

## Data Availability

The sequencing data supporting the findings of this study have been deposited in the NCBI Sequence Read Archive (SRA) under BioProject accession PRJNA1242245 and are publicy available.
