# Supplementary material for: Functional Diversity and Community Composition of Soil Fungi Associated With Canopy Dieback in Araucaria araucana Forests of Contrasting Edaphic Conditions
Source: Environ Microbiol Rep. 2026 Jun 23;18(3):e70361. doi: 10.1111/1758-2229.70361 (PMC13290381; doi:10.1111/1758-2229.70361)
Supplement: Supplementary file 1 — Table S1: Summary of sequencing read processing and quality filtering for all samples. [file EMI4-18-e70361-s001.docx]

**Supplementary Material**

Table S1. Summary of sequencing read processing and quality filtering for all samples.

| Sample ID | Raw reads (R1+R2) | Filtered reads | Denoised reads (DADA2) | Merged reads | Chimera-free reads | ASVs retained |
| --- | --- | --- | --- | --- | --- | --- |
| ASOIL_01 | 354,996 | 121,834 | 120,997 | 119,793 | 115,446 | 115,446 |
| ASOIL_02 | 282,785 | 132,919 | 132,243 | 129,947 | 128,127 | 128,127 |
| ASOIL_03 | 281,516 | 79,386 | 78,893 | 78,016 | 77,071 | 77,071 |
| ASOIL_04 | 304,320 | 190,923 | 190,023 | 188,260 | 185,753 | 185,753 |
| ASOIL_05 | 280,876 | 138,050 | 137,413 | 135,918 | 134,019 | 134,019 |
| ASOIL_06 | 220,682 | 116,900 | 116,356 | 114,485 | 105,228 | 105,228 |
| ASOIL_07 | 273,954 | 158,286 | 157,593 | 155,825 | 150,003 | 150,003 |
| ASOIL_08 | 194,954 | 134,170 | 133,337 | 131,041 | 124,511 | 124,511 |
| ASOIL_09 | 194,047 | 121,392 | 120,529 | 112,319 | 109,897 | 109,897 |
